# Case Report: A case of non-secretory multiple myeloma presenting with eosinophilia: diagnostic challenges and a focused literature review

**DOI:** 10.3389/fonc.2026.1833556

**Published:** 2026-04-22

**Authors:** Jirui Zhong, Xikun Liu, Jiduo Liu, Shanshan Xiao, Xuekui Gu, Zenghui Liu

**Affiliations:** 1 The First Clinical Medical School of Guangzhou University of Chinese Medicine, Guangzhou, China; 2 Department of Haematology, The First Affiliated Hospital of Guangzhou University of Chinese Medicine, Guangzhou, China

**Keywords:** bone marrow biopsy, eosinophilia, non-secretory multiple myeloma, osteolytic lesions, plasma-cell neoplasm

## Abstract

We report a rare case of non-secretory multiple myeloma (NSMM) presenting with marked eosinophilia and considerable diagnostic difficulty. A 56-year-old man was admitted with recurrent low back pain of more than 10 years’ duration that had rapidly worsened over the preceding 10 days, accompanied by diffuse pain and weight loss. Laboratory evaluation showed persistent eosinophilia, thrombocytopenia, elevated lactate dehydrogenase, and increased inflammatory markers. Imaging revealed multifocal osteolytic lesions involving the skull, vertebrae, ribs, and pelvis. However, serum and urine immunofixation electrophoresis were negative, qualitative urine Bence-Jones protein testing was negative, and repeated serum free light chain testing showed a normal κ/λ ratio, making the diagnosis particularly challenging. Bone marrow aspirate showed marked eosinophilia, and flow cytometry identified clonal plasma cells accounting for 6.1% of nucleated cells. Bone marrow biopsy with immunohistochemistry confirmed a plasma-cell neoplasm, while myeloma fluorescence *in situ* hybridization (FISH) revealed 1q21 gain/amplification together with chromosome 13-related abnormalities, including RB1 deletion and D13S319 abnormality. After exclusion of secondary and primary/clonal eosinophilic disorders, the patient was diagnosed with NSMM with eosinophilia. This case highlights the diagnostic challenge posed by the coexistence of marked eosinophilia and NSMM, which obscured the underlying plasma-cell malignancy despite repeatedly negative monoclonal protein studies. We also reviewed the limited literature on MM-associated eosinophilia to underscore the importance of integrating bone marrow findings, imaging, and cytogenetic evaluation in atypical cases.

## 1 Introduction

Multiple myeloma is a common clonal plasma-cell malignancy, accounting for over 10% of haematological malignancies (1). Among its subtypes, non-secretory multiple myeloma (NSMM) is rare and diagnostically challenging, accounting for approximately 3% of MM cases (2). It is characterised by minimal or absent secretion of monoclonal immunoglobulin in serum and urine, with a normal serum free light chain (sFLC) ratio (3). As a result, diagnosis often cannot rely on conventional monoclonal protein testing alone and instead requires bone marrow examination and ancillary studies. Although NSMM shares clinical manifestations with other MM subtypes, such as osteolytic bone lesions and haematopoietic suppression, cases presenting with peripheral blood eosinophilia are exceptionally uncommon (4). Such a presentation can complicate diagnosis because marked eosinophilia may obscure the underlying plasma-cell disorder, increasing the risk of misdiagnosis and delayed treatment. Here, we report a rare case of NSMM presenting with marked eosinophilia, describe the diagnostic challenges it posed, and review the relevant literature to improve clinical awareness of this unusual presentation.

## 2 Case presentation

On September 10, 2024, a 56-year-old man presented with recurrent low back pain of more than 10 years’ duration that had worsened markedly over the preceding 10 days. The pain had gradually intensified over the previous decade, but he had not undergone systematic evaluation or treatment and had relied only on intermittent oral analgesics for symptom relief. He had no known relevant past medical history and denied any relevant family or psychosocial history. During the current episode, the pain became substantially more severe and was accompanied by bilateral pain in the lumbar region, abdomen, and chest, making it difficult for him to stand or perform daily activities. He also reported an unintentional weight loss of 7.5 kg over the preceding month. After evaluation in our outpatient clinic, he was admitted to the Department of Bone and Soft Tissue Oncology with a preliminary diagnosis of unexplained bone destruction.

**Figure 1:**
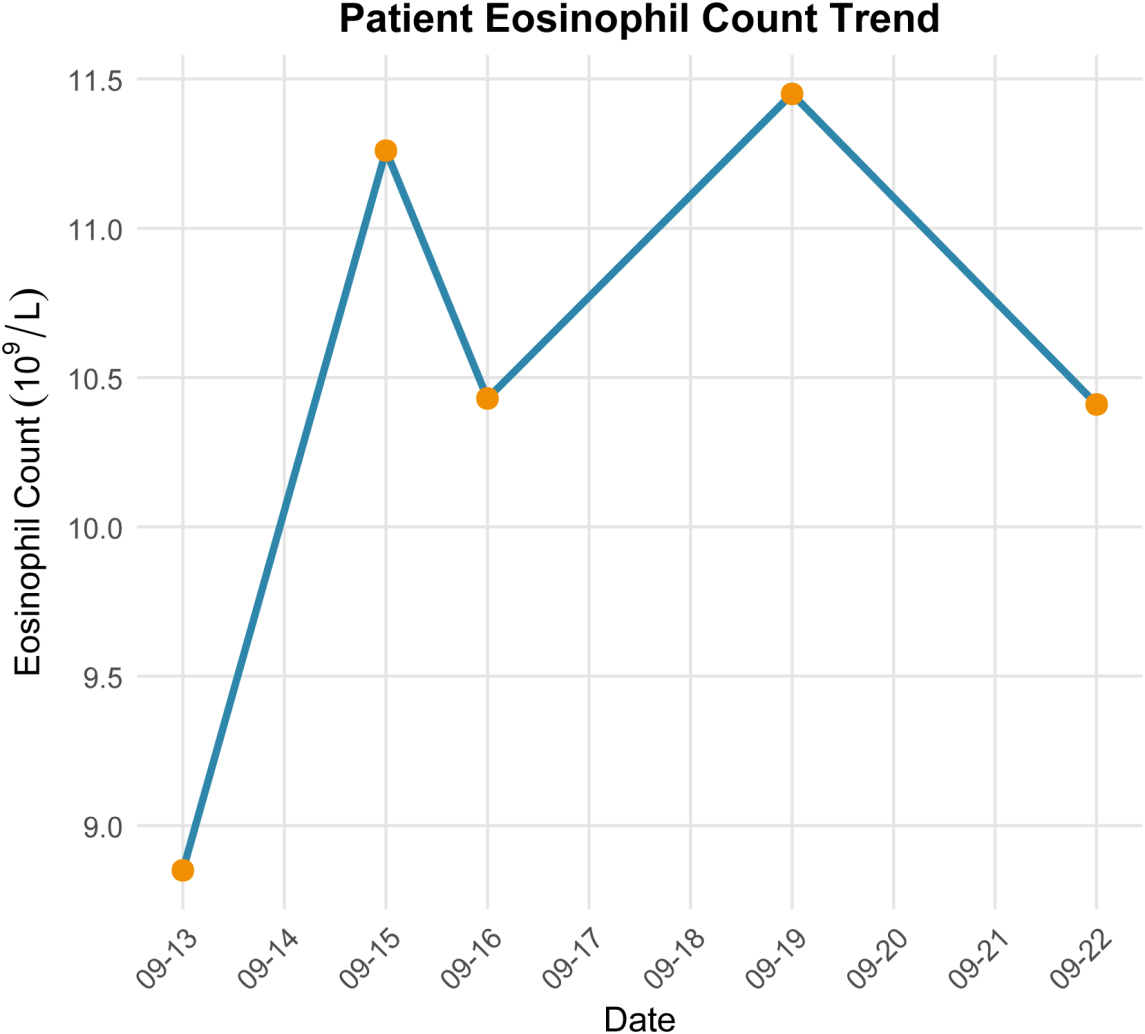
Dynamic changes in peripheral blood absolute eosinophil count (AEC) during hospitalisation.

Comprehensive laboratory and imaging evaluations were performed. Initial haematological testing showed a white blood cell count of 28.35 × 10^9^/L, red blood cell count of 4.12 × 10¹²/L, haemoglobin of 94 g/L, neutrophil count of 17.71 × 10^9^/L, eosinophil count of 8.85 × 10^9^/L, and platelet count of 47 × 10^9^/L. To further characterise the eosinophilia, serial haematological parameters were reviewed during hospitalisation. The absolute eosinophil count (AEC) was 8.85 × 10^9^/L on admission and remained persistently elevated during hospitalisation, ranging from 8.85 to 11.45 × 10^9^/L, confirming sustained eosinophilia (**Figure 1**). Peripheral blood smear examination confirmed marked absolute eosinophilia, with eosinophils accounting for approximately 36% of leukocytes. The eosinophils were predominantly mature, with segmented nuclei and typical eosinophilic granules, without dysplastic features or circulating blasts.

Biochemical and serological investigations were performed to evaluate the cause of the bone lesions and systemic symptoms. Total calcium was 2.59 mmol/L, lactate dehydrogenase (LDH) was 904 U/L, parathyroid hormone (PTH) was 0.8 pmol/L, and inorganic phosphorus was 1.61 mmol/L. Serum creatinine was 120 μmol/L, indicating mild renal impairment. Inflammation-related markers included C-reactive protein (CRP) of 21.0 mg/L, procalcitonin of 0.19 ng/mL, and serum amyloid A of 124 mg/L. No abnormalities were detected in ALT/AST, solid tumour markers, alkaline phosphatase, 25-hydroxyvitamin D, routine stool examination, or urinalysis. Serum and urine immunofixation electrophoresis were both negative for monoclonal protein. The serum free light chain (sFLC) assay, performed using high-sensitivity nephelometry, showed a normal κ/λ ratio of 0.65 (reference range, 0.26-1.65), with no detectable monoclonal free light chains. Twenty-four-hour urine light-chain testing was also negative. Ultrasonography of the liver, gallbladder, spleen, pancreas, portal venous system, kidneys, urinary tract, bladder, and prostate, together with echocardiography, did not reveal any space-occupying lesions.

Imaging was then reviewed to further clarify the nature of the skeletal lesions. Plain radiography demonstrated multifocal osteolytic lesions involving the skull, spine, ribs, and pelvis. Mild spinal degenerative changes were also present, but these findings did not account for the extent or distribution of the osteolytic lesions. Whole-body PET/CT would have been useful for assessing the full extent of skeletal involvement and for excluding possible extramedullary disease; however, the patient declined this examination because of financial constraints. Multiple myeloma was initially suspected. However, the diagnostic process was complicated by repeatedly negative monoclonal protein studies. Serum protein electrophoresis showed no detectable M protein, qualitative urine Bence-Jones protein testing was negative, and 24-hour urine light-chain testing was also negative. The serum free light chain κ/λ ratio remained within the normal range, whereas marked eosinophilia persisted. Given these conflicting findings, the Department of Haematology was consulted, and the patient was referred for further evaluation on September 12, 2024.

**Figure 2:**
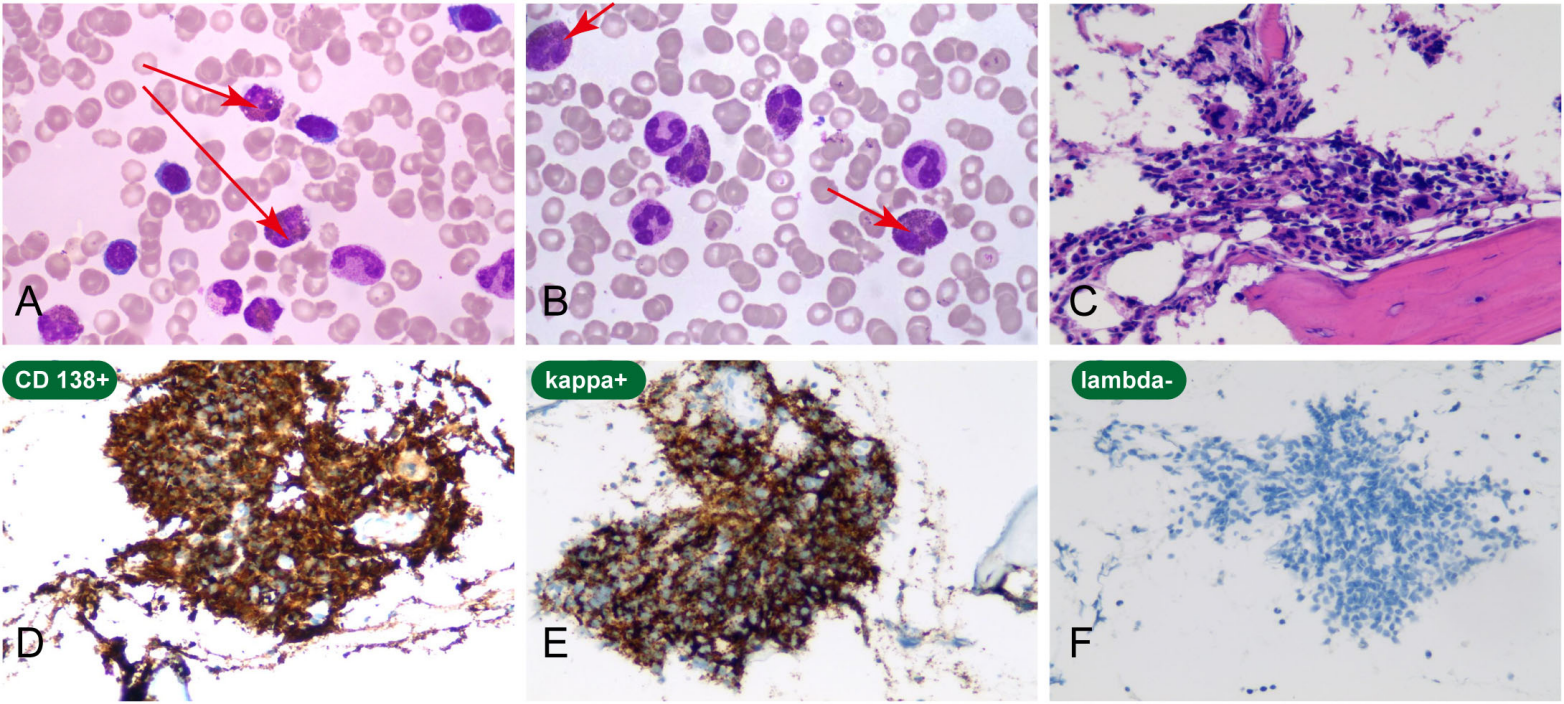
Cytological and histopathological findings in the present case. **(A)** Bone marrow aspirate smear showing eosinophilia (arrows). **(B)** Peripheral blood smear showing increased eosinophils with otherwise preserved distribution of other blood-cell lineages (arrows). **(C)** Haematoxylin and eosin staining of the bone marrow biopsy, showing cellular morphology and architectural features (arrows). **(D)** Immunohistochemical staining for CD138, showing positive expression in the plasma-cell population. **(E)** Kappa staining, showing positive expression in the plasma-cell population. **(F)** Lambda staining, showing negative expression in the corresponding plasma-cell population. Original magnification: all panels, ×200.

Bone marrow aspiration and biopsy were performed on September 13, 2024. The bone marrow aspirate smear was of satisfactory quality and showed active marrow proliferation, with 200 nucleated cells counted for differential analysis. Granulopoiesis was hyperplastic, with a marked increase in eosinophils and eosinophil precursors at all stages, accounting for 48% of nucleated cells. Erythropoiesis was hypocellular but morphologically unremarkable. Megakaryocytes were not identified on the aspirate smear. Although this finding may be relevant to the severe thrombocytopenia (platelet count, 47 × 10^9^/L), it was interpreted cautiously because aspirate assessment can be affected by sampling variability. Given the limited biopsy tissue, megakaryopoiesis could not be adequately evaluated, and no definitive conclusion regarding the cause of thrombocytopenia could be drawn. Morphological examination showed 2.5% plasma cells, some with immature features. Flow cytometry of the bone marrow aspirate identified a population of monoclonal plasma cells accounting for 6.1% of total nucleated cells, with an immunophenotype of CD38+, CD138+, partial CD56+, and κ light-chain restriction. The bone marrow core biopsy was obtained in a routine and technically adequate manner and was considered diagnostically informative on pathological review. Histopathological evaluation showed increased plasma-like cells. Immunohistochemistry demonstrated CD138 and MUM1 positivity with κ light-chain restriction (κ positive, λ negative), supporting the presence of a clonal plasma-cell neoplasm. Because the biopsy tissue was limited, a detailed assessment of eosinophilic infiltration pattern in the core biopsy was not possible. In the context of flow cytometric evidence of a κ-restricted clonal plasma-cell population, biopsy-confirmed plasma-cell neoplasm, and myeloma-defining clinical features, the biopsy findings did not materially weaken the overall diagnostic confidence. No significant marrow fibrosis was observed (**Figure 2**).

To exclude secondary causes of eosinophilia, a detailed medication history was reviewed. There was no recent use of antibiotics, non-steroidal anti-inflammatory drugs, or anticonvulsants, all of which may induce eosinophilia (5). The patient also denied any history of atopic disease, including asthma, allergic rhinitis, or eczema. Infectious and autoimmune causes were investigated systematically. Stool examination showed no ova or parasites. Serum IgE was within the normal range. In addition, parasitic serological testing was performed, and a seven-item parasite antibody panel was negative, making parasitic infection less likely. After reactive eosinophilia had been excluded on clinical and laboratory grounds, primary/clonal eosinophilia was considered. Genetic testing was therefore performed and was negative for rearrangements involving FGFR1, PDGFRB, and JAK2, as well as for FIP1L1-PDGFRA and ETV6-PDGFRA fusion genes.

Myeloma-directed FISH testing subsequently showed 1q21 gain/amplification, RB1 deletion, and D13S319 abnormality, whereas del(17p)/TP53 deletion was not detected. The myeloma FISH panel used in our centre also assessed recurrent high-risk abnormalities such as t(4;14) and t(14;16), neither of which was identified in this patient. Oligosecretory myeloma was considered excluded on the basis of the available laboratory evidence, because repeated serum free light-chain testing showed a persistently normal κ/λ ratio without detectable monoclonal free light chains, while serum and urine immunofixation electrophoresis and 24-hour urine light-chain testing also remained negative. Taken together, these concordant negative findings did not support oligosecretory disease in the present case. Taken together, the imaging findings, bone marrow flow cytometry and biopsy results, and the overall clinical picture supported the diagnosis of active myeloma despite repeatedly negative serum and urine monoclonal protein studies. The patient was ultimately diagnosed with non-secretory multiple myeloma with eosinophilia, with cytogenetic abnormalities including 1q21 gain/amplification and chromosome 13-related lesions, namely RB1 deletion and D13S319 abnormality, suggestive of adverse-risk disease biology.

Following diagnosis, immediate anti-myeloma chemotherapy was recommended. The proposed initial regimen at our centre was bortezomib, lenalidomide, and dexamethasone (VRd). This regimen was selected because the patient had newly diagnosed active myeloma with myeloma-defining clinical features and required prompt disease control. In addition, given the patient’s financial constraints, a more economically accessible triplet regimen was considered more practical in this setting. Although daratumumab-containing quadruplet regimens have emerged as important frontline options in newly diagnosed multiple myeloma, their additional value in this rare presentation of eosinophilia-associated NSMM remains uncertain, and no daratumumab-containing regimen was identified among the eosinophilia-associated cases included in our focused review. The urgency of treatment was emphasised because of the active disease status and rapid clinical progression. During hospitalisation, the patient received supportive management, including analgesia, hydration, and correction of electrolyte and metabolic abnormalities; however, definitive anti-myeloma chemotherapy was not initiated before discharge. After discussion with his family, the patient requested discharge in order to continue treatment at a local hospital and was discharged on September 22, 2024. Despite repeated recommendations for prompt standard anti-myeloma therapy, he did not subsequently receive treatment because of financial constraints. During follow-up, the family reported progressive clinical deterioration, including worsening bone pain, declining mobility, and worsening general condition. No interval anti-myeloma treatment was documented. Approximately 6 months after discharge, follow-up communication with the family confirmed the patient’s death, reportedly due to progression of NSMM.

The clinical timeline of this case is summarized in **Table 1**. To clarify the diagnostic process and demonstrate fulfilment of the 2014 International Myeloma Working Group (IMWG) criteria for NSMM (3), the key findings are summarised in **Table 2**.

**Table 1:** Timeline.

Date	Event
>10 years before admission	Recurrent low back pain, managed intermittently with oral analgesics
10 days before admission	Marked worsening of pain
September 10, 2024	Admission for unexplained bone destruction
September 12, 2024	Referral to the Department of Haematology for further evaluation
September 13, 2024	Bone marrow aspiration and biopsy performed
During hospitalisation	Repeatedly negative serum/urine monoclonal protein studies; persistent eosinophilia; myeloma-directed FISH performed
During hospitalisation	VRD regimen recommended, but definitive anti-myeloma therapy was not initiated before discharge
September 22, 2024	Discharged to continue treatment at a local hospital
After discharge	No anti-myeloma treatment received because of financial constraints
Approximately 6 months after discharge	Family confirmed death, reportedly due to progression of NSMM

**Table 2:** Diagnostic findings supporting non-secretory multiple myeloma based on the 2014 international myeloma working group framework.

Diagnostic element	Patient findings	Supports NSMM
Serum/urine M protein	Negative by immunofixation electrophoresis	Yes
Serum free light chain (sFLC)	Normal κ/λ ratio (0.65); no monoclonal free light chains	Yes
24-hour urine light chain	Negative	Yes
Bone marrow clonal plasma cells	6.1% by flow cytometry (κ light chain restriction); biopsy confirmed plasma-cell neoplasm	Yes
CRAB features	Hypercalcaemia (2.59 mmol/L), renal impairment (Cr 120 μmol/L), anaemia (Hb 94 g/L), osteolytic lesions	Yes

## 3 Discussion

We report a rare case of non-secretory multiple myeloma (NSMM) presenting with marked eosinophilia. The key findings supporting the diagnosis of NSMM according to the 2014 International Myeloma Working Group (IMWG) framework are summarised in **Table 2**. In this case, oligosecretory myeloma was considered excluded on the basis of the available laboratory evidence, because repeated serum free light-chain testing showed a persistently normal κ/λ ratio without detectable monoclonal free light chains, while serum and urine immunofixation electrophoresis and 24-hour urine light-chain testing also remained negative. Taken together, these concordant negative results did not support even a low-level secretory phenotype and therefore favoured NSMM. We also acknowledge that histological assessment of the core biopsy had limitations and that sampling variation may have affected precise estimation of the overall marrow plasma-cell burden; however, the specimen was not considered non-diagnostic. However, repeat biopsy or additional tissue sampling was not pursued in the present case because the diagnosis was already considered sufficiently supported by concordant evidence from bone marrow flow cytometry demonstrating a κ-restricted clonal plasma-cell population, biopsy immunohistochemistry confirming a plasma-cell neoplasm, and myeloma-defining clinical and radiological features, particularly multifocal osteolytic lesions together with anaemia and hypercalcaemia. Thus, diagnostic confidence rested on the integration of pathology, flow cytometry, imaging, and the overall clinical picture, rather than on marrow plasma-cell percentage alone. The creatinine elevation was modest and not specific for myeloma and was therefore interpreted as a supportive abnormality rather than a stand-alone myeloma-defining event.

Eosinophilia may be inherited, secondary/reactive, primary/clonal, or idiopathic, and careful exclusion of these categories was essential in the present case (6). The absence of a family history made hereditary eosinophilia unlikely. Further evaluation did not support parasitic, autoimmune, or allergic causes, making secondary eosinophilia unlikely. Parasitic infection was considered unlikely based on the absence of suggestive exposure history, negative stool examination, normal serum IgE, and a negative seven-item parasite antibody panel. In addition, molecular testing for primary/clonal eosinophilic disorders was negative. An exclusive focus on eosinophilia, without attention to marrow plasma-cell abnormalities, might have led to an incorrect diagnosis of isolated eosinophilic disease.

To place the present case in context, we performed a focused review of published reports on multiple myeloma (MM) or plasma-cell neoplasms associated with eosinophilia. PubMed was searched up to January 1, 2026, using combinations of the terms “multiple myeloma,” “plasma cell myeloma,” “non-secretory multiple myeloma,” “eosinophilia,” “hypereosinophilia,” and “plasma cell dyscrasia.” Reference lists of relevant articles were also screened manually. English-language case reports and case series with sufficient clinical detail to support a diagnosis of MM or plasma-cell neoplasm associated with eosinophilia were included. Reports in which eosinophilia was temporally related to anti-myeloma therapy were retained but analysed separately. Duplicate reports, conference abstracts without adequate clinical detail, and articles with insufficient comparative information were excluded.

MM is a clonal plasma-cell malignancy accounting for just over 10% of haematological malignancies (3, 7). NSMM comprises approximately 3% of MM cases and is characterised by minimal or absent secretion of monoclonal immunoglobulin (4, 8). Because NSMM lacks readily detectable monoclonal protein in serum and urine, diagnosis may be delayed when clinicians rely too heavily on conventional M-protein studies. In the present case, marked eosinophilia further obscured the underlying plasma-cell disorder and increased diagnostic complexity. Because NSMM shares many clinical features with secretory MM, diagnosis requires integration of clinical, radiological, pathological, and cytogenetic findings. To reduce misdiagnosis, challenging cases may benefit from multidisciplinary review and greater clinician awareness of rare NSMM presentations.

The cytogenetic findings in this case also merit attention. The detected 1q21 gain/amplification is generally considered an adverse prognostic feature in multiple myeloma and has been associated with more aggressive disease biology and inferior survival in many cohorts (9, 10). Chromosome 13-related abnormalities (including RB1 deletion and D13S319 abnormality) may also indicate unfavourable disease behaviour, although their independent prognostic impact can vary depending on coexisting lesions and the risk stratification model used. Overall, the cytogenetic profile suggested biologically aggressive disease, which was also clinically consistent with extensive osteolytic lesions, thrombocytopenia, elevated LDH, and rapid progression. At present, these cytogenetic abnormalities should be regarded as markers of aggressive myeloma biology rather than established drivers of eosinophilia.

The coexistence of myeloma and eosinophilia is uncommon and remains poorly characterised. Our focused review identified only a small number of reported cases of MM or related plasma-cell neoplasms associated with eosinophilia, with the exact count varying according to inclusion criteria and the availability of sufficient clinical details (11–14). No prior case of non-secretory multiple myeloma with eosinophilia was identified in our focused review. The published cases summarised in **Table 3** suggest substantial heterogeneity in the timing of eosinophilia, clinical presentation, treatment exposure, and outcomes, highlighting the need for cautious comparison and mechanistic interpretation.

**Table 3:** Reported cases of multiple myeloma associated with eosinophilia and comparison with the present case.

Case	Reference	Age/Sex	AEC (×10^9^/L)	Association	Myeloma subtype/CRAB	Treatment	Outcome
1	Sekiguchi et al (11)	78/M	2.35	Tx-related (LEN)	IgG/NA	MP → VD → RD	Eos improved after LEN withdrawal; PR, died
2	Glantz et al (12)	49/M	109.7	MM-associated at Dx	IgGλ/NA	Steroids + CT	Eos decreased; clinical improvement
3	Oka et al (13)	85/F	13.5	MM-associated at Dx	IgGκ/R/A/B	VRD	Eos improved; VGPR
4	Lee et al (14)	31/M	4.31	MM-associated at Dx	IgGλ + IgGκ/A	Dex → VD → ASCT	Eos improved; CR
5	Present case	56/M	8.85	MM-associated at Dx	Non-secretory/C/R/A/B	Supportive care only (VRD recommended)	Eos persistent/progressive; died

AEC, absolute eosinophil count; Dx, at diagnosis; Tx, during treatment; LEN, lenalidomide; CRAB, hypercalcaemia (C), renal impairment (R), anaemia (A), and bone lesions (B); PR, partial response; VGPR, very good partial response; CR, complete response; VRD, bortezomib + lenalidomide + dexamethasone; VD, bortezomib + dexamethasone; RD, lenalidomide + dexamethasone; ASCT, autologous stem cell transplantation; MP, melphalan + prednisolone; Dex, dexamethasone; CT, chemotherapy; NA, not available.

As summarized in **Table 3**, comparison with previously reported cases suggests substantial heterogeneity in the clinical presentation, eosinophil burden, and outcomes of MM-associated eosinophilia. In some reports, eosinophilia was present at or near the time of MM diagnosis and improved after anti-myeloma therapy, supporting a possible paraneoplastic or disease-associated process; in contrast, other reports described eosinophilia developing during treatment (particularly lenalidomide exposure), favouring a treatment-related mechanism. Reported manifestations range from isolated eosinophilia to severe multi-organ involvement or inflammatory/paraneoplastic features. In our case, eosinophilia was present before anti-myeloma therapy, and extensive evaluation did not support allergic, parasitic, autoimmune, or primary/clonal eosinophilic disorders, favouring a disease-associated MM process. Notably, unlike most previously reported secretory MM cases, our patient had repeatedly negative serum/urine monoclonal protein studies and a normal serum free light-chain ratio, which markedly increased diagnostic complexity.

Because treatment exposure differed substantially across reported cases, direct outcome comparison is limited. Several previously reported patients received anti-myeloma therapy (and, in some cases, autologous stem cell transplantation), with improvement in eosinophilia and/or myeloma burden reported after treatment, whereas our patient did not receive standard anti-myeloma therapy after discharge because of financial constraints and died approximately 6 months later. Therefore, the unfavourable outcome in our case should not be attributed solely, or even predominantly, to adverse disease biology. Rather, it was likely shaped by multiple interacting factors, among which the absence of timely definitive anti-myeloma treatment was a major contributor, together with aggressive disease features and diagnostic delay related to the atypical NSMM presentation. Because no definitive anti-myeloma treatment was actually administered, the present case provides limited management-related insight beyond highlighting the consequences of delayed or absent therapy in an aggressive presentation. In addition, follow-up information was obtained mainly through family communication, and the available outcome data were limited, which restricts a more detailed analysis of disease progression.

With respect to pathophysiology, previously reported cases suggest that eosinophilia in MM may be treatment-related in some patients, such as lenalidomide-associated eosinophilia, whereas in others it may represent a disease-associated reactive or paraneoplastic process (15). In the present case, after systematic exclusion of familial, secondary, and primary/clonal eosinophilic disorders, the eosinophilia was considered most likely reactive to the underlying plasma-cell neoplasm, possibly as a paraneoplastic phenomenon. One possible explanation is remodelling of the bone marrow microenvironment accompanied by cytokine dysregulation in MM, as interactions between myeloma cells and marrow stromal cells may promote the release of growth factors, chemokines, and inflammatory mediators, including IL-6, IL-8, IL-10, and TNF, which may influence both tumour-cell survival and normal haematopoietic progenitors (16, 17). Consistent with this possibility, our patient showed marked marrow eosinophilia together with clonal plasma-cell proliferation. However, these proposed cytokine-mediated mechanisms remain speculative in the present case because no cytokine profiling or functional validation was performed.

Previous studies provide biological context supporting the plausibility of this hypothesis, although they fall short of case-specific mechanistic proof. Eosinophils have been implicated in tissue remodelling and tumour-associated inflammatory responses, and the bone marrow microenvironment is a key regulator of both haematopoietic and immune-cell homeostasis (18, 19). In our patient, the cytogenetic profile, including 1q21 gain/amplification, RB1 deletion, and D13S319 abnormality, may reflect more aggressive myeloma biology (9, 10). It is therefore biologically plausible that a more dysregulated or inflammatory marrow microenvironment in this setting could favour eosinophil expansion through cytokine networks and downstream signalling pathways, including IL-3/IL-5-related signalling and JAK-STAT activation (20–22). Nevertheless, these mechanisms were not directly examined in the present case, and the coexistence of eosinophilia and cytogenetic abnormalities should be interpreted as a hypothesis-generating association rather than evidence of a direct causal relationship.

Given the exploratory nature of these proposed mechanisms, further studies are needed to clarify the pathophysiological relationship between MM and eosinophilia in cases such as this. Prospective collection of similar cases, together with standardised cytokine profiling (e.g., IL-3, IL-5, and GM-CSF), bone marrow microenvironment characterisation, and longitudinal monitoring of eosinophil counts in relation to tumour burden and treatment response, would be particularly informative. Integration of cytogenetic findings with molecular analyses may also help distinguish reactive eosinophilia, treatment-related eosinophilia, and eosinophilia associated with underlying disease biology. Such efforts may improve mechanistic understanding and refine the diagnostic interpretation of eosinophilia in MM. In accordance with the CARE guidelines, a formal patient perspective was not available because the patient died during follow-up and no first-person statement could be obtained at the time of manuscript preparation.

## 4 Conclusion

In conclusion, this case highlights the diagnostic difficulty posed by non-secretory multiple myeloma presenting with eosinophilia, particularly when monoclonal protein studies are repeatedly negative. In patients with eosinophilia accompanied by bone pain, anaemia, thrombocytopenia, renal dysfunction, or osteolytic lesions, clinicians should maintain a high index of suspicion for an underlying plasma-cell neoplasm. In addition to routine laboratory and imaging evaluation, bone marrow aspiration and biopsy, together with myeloma-directed FISH testing, may be critical for establishing the diagnosis. Early recognition of such atypical presentations may reduce diagnostic delay and facilitate timely treatment.

## Data Availability

The original contributions presented in the study are included in the article/supplementary material. Further inquiries can be directed to the corresponding authors.
